# Unraveling the Excellent High-Temperature Oxidation Behavior of FeNiCuAl-Based Alloy

**DOI:** 10.3390/ma18153679

**Published:** 2025-08-05

**Authors:** Guangxin Wu, Gaosheng Li, Lijun Wei, Hao Chen, Yujie Wang, Yunze Qiao, Yu Hua, Chenyang Shi, Yingde Huang, Wenjie Yang

**Affiliations:** 1 School of Materials Science and Engineering, Zhengzhou University, Zhengzhou 450001, Chinachen_yang_shi@csu.edu.cn (C.S.); 2 State Power Investment Corporation Limited Central Research Institute, Beijing 102209, China; ligaosheng@spic.com.cn (G.L.); weilijun@spic.com.cn (L.W.)

**Keywords:** high-entropy alloys, high-temperature oxidation resistance, corrosion resistance

## Abstract

This study synthesized FeNiCuAlX high-entropy alloys (HEAs) (where X = Cr, Co, Mn) using arc melting and investigated their high-temperature oxidation behavior in air at 900 °C. The oxidation kinetics of all alloys followed a parabolic rate, with the oxidation rate constants (kp) of FeNiCuAlCr, FeNiCuAlCo, and FeNiCuAlMn being approximately two to three orders of magnitude lower than that of the FeNiCu alloy. Specifically, FeNiCuAlCr exhibited the lowest kp value of 1.72 × 10^−6^ mg^2^·cm^4^/s, which is significantly lower than those of FeNiCuAlCo (3.29 × 10^−6^ mg^2^·cm^4^/s) and FeNiCuAlMn (1.71 × 10^−5^ mg^2^·cm^4^/s). This suggests that the addition of chromium promotes the formation of a dense Al_2_O_3_/Cr_2_O_3_ oxide layer, significantly enhancing the oxidation resistance. Furthermore, corrosion resistance was assessed through potentiodynamic polarization and electrochemical impedance spectroscopy in a 3.5% NaCl solution. FeNiCuAlCr demonstrated exceptional resistance to localized corrosion, as indicated by its low corrosion current density (45.7 μA/cm^2^) and high pitting potential (−0.21 V), highlighting its superior corrosion performance.

## 1. Introduction

Since ancient times, human civilization has been constantly striving to develop advanced materials, explore new metals, and innovate alloys, which has been crucial for technological evolution over thousands of years [1]. Traditional alloys are typically based on a single primary element, a practice that somewhat limits their compositional diversity and the potential for new properties. Therefore, there is an urgent need to explore unconventional alloys to meet the growing demand for enhanced material performance [2].

High-entropy alloys (HEAs) are materials composed of five or more principal metallic elements in nearly equimolar ratios, typically forming one or more solid solution phases [3]. About a decade ago, Cantor et al. first synthesized this type of alloy, producing a five-component Fe_20_Cr_20_Mn_20_Ni_20_Co_20_ alloy using melt spinning. In this high-entropy alloy, the five transition metal elements exhibited a high degree of compatibility, successfully forming a single face-centered cubic (FCC) solid solution [4]. Meanwhile, Yeh et al. reported several HEAs produced by arc melting, consisting of elements such as Cu, Ni, Ti, and Cr. These alloys were termed “high-entropy alloys,” a name that reflects the high configurational entropy resulting from the random mixing of the different elements [5]. Today, HEAs have become a hot topic in materials science research. However, due to the relatively late start of HEA research, the field is still in its early stages of development, and compared with the well-established research on conventional alloys, many challenges remain to be addressed [6].

Medium-entropy alloys (MEAs) and high-entropy alloys (HEAs) are frequently utilized in high-temperature and corrosion-resistant applications, with HEAs being particularly notable. HEAs exhibit a range of distinctive and desirable properties, making them highly suitable for various engineering applications, including those requiring exceptional hardness [7], damage tolerance [8], good wear resistance [9] and remarkable corrosion and oxidation resistance [10,11,12], For instance, the oxidative behavior of the AlCoCrFeNi HEA at 1100 °C was investigated in 2021 by Lu. [13] who observed that this alloy demonstrates an exceptionally low oxidation rate and exhibits strong resistance to oxide scale spallation. Similarly, Butler [14] studied the high-temperature oxidation behavior of AlxCrCoNiFe(Si). Their findings indicated that when the aluminum content (atomic fraction) reached 10%, the oxidation kinetics at 1050 °C adhered to a parabolic rate law [15]. Specifically, the oxidation rates for the alloys with lower and higher Al contents were 1.1 × 10^−11^ and 5.1 × 10^−15^ (g^2^/cm^4^ s). The superior oxidation resistance observed at high temperatures was attributed to the microstructural characteristics: low-Al alloys formed a discontinuous Cr_2_O_3_ outer layer and Al_2_O_3_ substrate containing AlN, whereas high-Al alloys developed a continuous Al_2_O_3_ layer without an inner oxide layer. Furthermore, Liu [16] demonstrated that CoCrFeNiAlx HEAs (x = 0.15, 0.4) exhibited superior antioxidant properties compared with HR3C steel in supercritical water at 550 °C and 600 °C. Their research emphasized the significant influence of aluminum content on the oxidation resistance of HEAs. Collectively, these findings suggest that, similar to conventional alloys, HEAs undergo selective oxidation, with aluminum and chromium playing crucial roles in determining the oxidation behavior [17,18].

Previous studies have demonstrated that inert anodes used in aluminum electrolysis, made from medium-entropy alloys (Fe-Ni-Cu), exhibit excellent high-temperature stability and corrosion resistance. These alloys form a protective NiFe_2_O_4_ layer at elevated temperatures, significantly improving their corrosion resistance [19,20]. Building on this, this research aims to investigate the high-temperature oxidation behavior of FeNiCuAlCr, FeNiCuAlCo, and FeNiCuAlMn high-entropy alloys. The use of Cr, Co, and Mn in high-entropy alloys (HEAs) aims to enhance the various properties of the alloy. Cr primarily improves the alloy’s corrosion resistance and oxidation resistance by forming a stable protective oxide layer. Co enhances the high-temperature stability of the alloy and increases its strength, making it suitable for high-stress and high-temperature environments. Mn helps to improve the phase stability of the alloy and enhances its thermal stability. Specifically, the alloys were synthesized through arc melting from high-purity raw materials, and their oxidation behavior at 900 °C in air was analyzed. The objective of this study is to understand the effects of individual alloying elements on the oxidation kinetics and the oxide formation processes. We have also examined the microstructure of the oxide layers to provide insights into their formation mechanisms. Despite the growing body of research on the oxidation behavior of high-entropy alloys, there is a significant gap in the study of the oxidation performance of these specific FeNiCuAl-based alloys at 900 °C. Moreover, the corrosion resistance of these alloys in a 3.5% NaCl solution was evaluated using potentiodynamic polarization and electrochemical impedance spectroscopy. This study aims to fill this gap by providing a detailed understanding of their oxidation and corrosion behaviors, which are crucial for their application in harsh environments.

## 2. Experimental Procedures

### 2.1. Material Preparation

The high-entropy alloy was synthesized via arc melting under a high-purity argon atmosphere (99.999%) (Yuanzheng Special Gas Company, Xinxiang, China). The alloy was prepared using a mixture of highly pure raw materials, including Fe (99.99 wt%), Ni (99.99 wt%), Cu (99.99 wt%), Al (99.99 wt%), Cr (99.99 wt%), Co (99.99 wt%), and Mn (99.99 wt%). All of the high-purity metal powders were purchased from Macklin Company (Shanghai, China). To ensure alloy homogeneity, the button was flipped and the alloys were re-melted at least three times during the arc melting process.

### 2.2. Isothermal Oxidation Testing

Samples (10 mm × 10 mm × 10 mm) were cut from the alloys using electric discharge machining and ground to a 2000-grit finish with silicon carbide paper. Subsequently, the samples were ultrasonically cleaned in acetone (Sinopharm Chemical Reagent Company, Shanghai, China). Isothermal oxidation tests were conducted in a box furnace (Henan Synthe Corporation, Zhengzhou, China) at 900 °C for durations of 2, 12, 36, 48, 60, 84, and 108 h in air. After furnace cooling to room temperature, the weight of each sample was measured. Each experiment was performed in triplicate for each alloy, and the average mass gain was calculated to determine the oxidation kinetics.

### 2.3. Corrosion Behavior in 3.5% NaCl Solution

**Figure 1:**
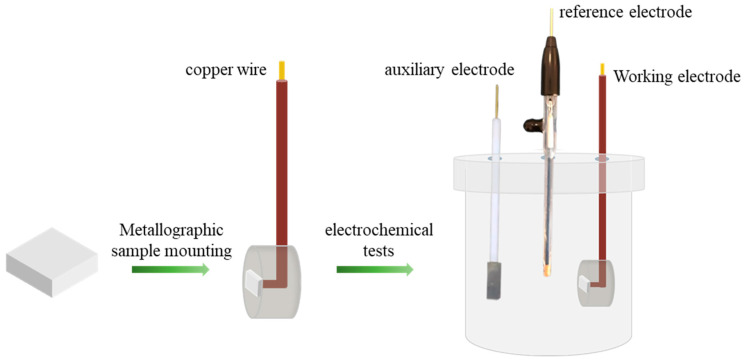
Flowchart of the corrosion behavior test in 3.5% NaCl solution.

The sample size for the electrochemical tests was 10 mm × 10 mm × 2 mm. Each sample was welded to a copper wire (Aladdin Scientific Corp, Shanghai, China) and embedded in epoxy resin (Aladdin Scientific Corp, Shanghai, China), leaving a 10 mm × 10 mm working surface exposed (Figure 1). The electrochemical experiments were performed using an electrochemical workstation (Princeton Applied Research, Berwyn, IL, USA). The high-entropy alloy was employed as the working electrode, with a platinum mesh as the auxiliary electrode, and a saturated calomel electrode (SCE) serving as the reference electrode. A 3.5% NaCl solution was utilized as the electrolyte. Before commencing the tests, the samples were submerged in the electrolyte for one hour to ensure a stable state. Electrochemical impedance spectroscopy (EIS) was conducted at the open circuit potential (OCP), spanning a frequency range from 10^6^ Hz to 10^−2^ Hz, with an amplitude of 10 mV. Following this, potentiodynamic polarization (PDP) curves were obtained within a voltage window of −0.75 to 2 V (relative to OCP), using a scan rate of 1 mV/s. To guarantee the consistency and reliability of the results, each experimental group was tested in triplicate.

### 2.4. Characterization Techniques

The phase composition of the high-entropy alloy was characterized using X-ray diffraction (Rigaku SmartLab SE, Rigaku Corporation, Tokyo, Japan), employing Cu-Kα radiation. The analysis was conducted with a scanning rate of 2°/min, a step increment of 0.02°, and operational settings of 40 kV for the accelerating voltage and 40 mA for the current. The diffraction data were collected within the angular range of 10° to 90°. To evaluate the surface and cross-sectional features of the high-entropy alloy following isothermal oxidation, scanning electron microscopy (SEM, ZEISS Sigma300, ZEISS, Oberkochen, Germany) was employed, integrated with an energy-dispersive spectrometer (EDS) (Bruker, Karlsruhe, Germany) for detailed elemental analysis. The XANES with soft X-rays were collected at the National Synchrotron Radiation Laboratory (NSRL).

## 3. Results and Discussion

### 3.1. Substrate Analysis

**Figure 2:**
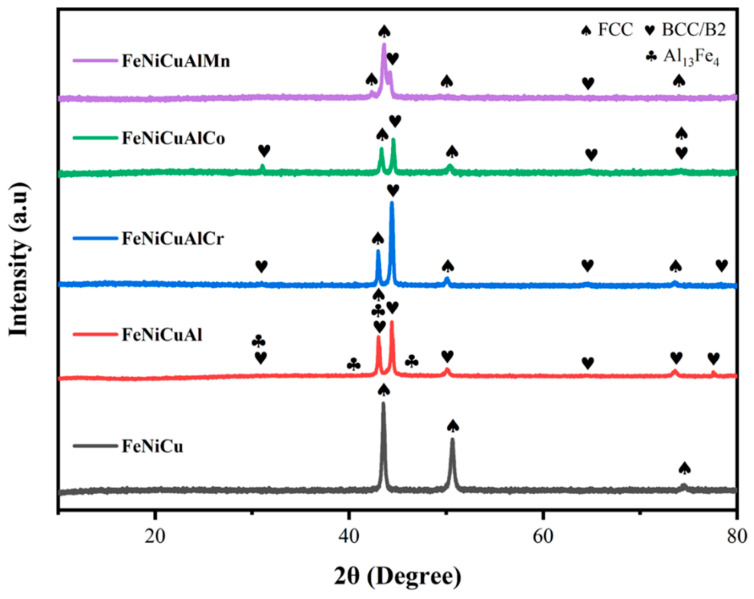
XRD patterns of as-cast FeNiCu, FeNiCuAl alloy, FeNiCuAlCr, FeNiCuAlCo, and FeNiCuAlMn HEAs.

**Figure 3:**
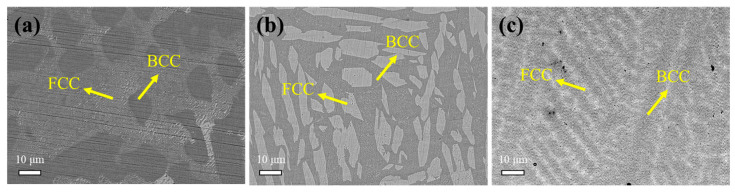
SEM image (BSE) of the as-cast HEAs: (**a**) FeNiCuAlCr; (**b**) FeNiCuAlCo; (**c**) FeNiCuAlMn.

The XRD patterns of the FeNiCu and FeNiCuAl alloys, as well as the FeNiCuAlCr, FeNiCuAlCo, and FeNiCuAlMn high-entropy alloys (HEAs) in their as-cast states following re-melting are shown in Figure 2 and Figure 3. The XRD analysis reveals that the FeNiCuAlCr, FeNiCuAlCo, and FeNiCuAlMn HEAs exhibit a dual-phase structure comprising face-centered cubic (FCC) and body-centered cubic (BCC) phases. In contrast, the FeNiCu alloy displays a single-phase FCC structure. The FeNiCuAl alloy, however, exhibits a multiphase structure in its XRD spectrum, which includes FCC and BCC phases alongside a notable presence of the Al_13_Fe_4_ phase (space group C2/m, monoclinic lattice). The Al_13_Fe_4_ phase, belonging to the orthorhombic crystal system, acts as an intermediate product between aluminum (Al) and iron (Fe). This intermetallic compound is known for its low mechanical strength and high brittleness, which can lead to crack formation within the alloy matrix [21]. Furthermore, as shown in Figure S1, the phase distribution of the FeNiCuAl alloy is uneven, with significant segregation. This inhomogeneity can lead to localized variations in the material’s properties, thereby weakening its overall mechanical strength and stability, making it unsuitable for high-performance applications. Given these characteristics, it is hypothesized that the FeNiCuAl alloy may demonstrate an inferior performance compared with the high-entropy alloys. Moreover, as the focus of this study is on high-entropy alloys containing five or more principal elements, the FeNiCuAl alloy will not be considered in subsequent analyses. After the addition of Al, a mixed structure of FCC and BCC phases can appear. The addition of Cr, Co, or Mn to FeNiCu and FeNiCuAl alloys significantly affects the phase formation of high-entropy alloys. Cr tends to stabilize the FCC structure, especially in high-entropy alloys. Cr has a high solubility in Fe and Ni, which can enhance the stability of the FCC phase. Co also helps stabilize the FCC structure and can maintain the coexistence of both FCC and BCC phases; however, under certain conditions, it may promote phase separation. Mn generally stabilizes the BCC phase in alloys and, under specific temperature and compositional conditions, may promote the formation of a dual-phase structure. The atomic arrangement in the dual-phase FCC/BCC structure plays a crucial role in the overall performance of the material. Compared with single-phase materials, dual-phase structures typically exhibit better thermal stability at higher temperatures, as the BCC phase is usually more stable than the FCC phase at elevated temperatures. The coexistence of both FCC and BCC phases enhances the mechanical properties of the alloy through mechanisms like solid solution strengthening and phase boundary strengthening. The dual-phase structure can also impact the alloy’s corrosion resistance, making it suitable for harsh environments.

At the microscopic scale, the as-cast alloys displayed large, coarse dendritic structures with varying levels of development. In each case, this dendritic formation was associated with the segregation of cubic solid phases [22]. Scanning electron microscopy (SEM) backscatter electron imaging (Figure 3) revealed dendritic segregation [23]. Overall, the microstructure was primarily characterized by dendritic and interdendritic regions. The interdendritic phase had a body-centered cubic (BCC) structure, while the dendritic phase had a face-centered cubic (FCC) structure.

### 3.2. Oxidation Kinetics

**Figure 4:**
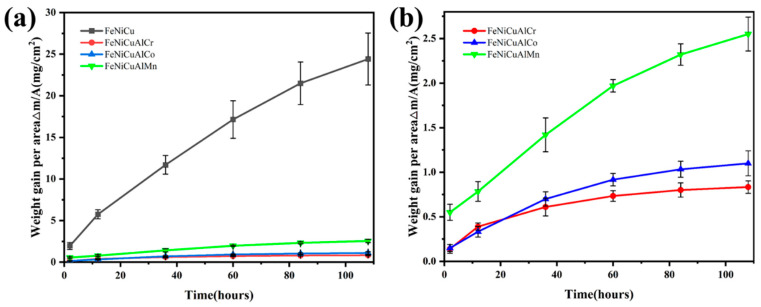
The mass gain plot of the (**a**) FeNiCu alloy and FeNiCuAlCr, FeNiCuAlCo, and FeNiCuAlMn HEAs and (**b**) the FeNiCuAlCr, FeNiCuAlCo, and FeNiCuAlMn HEAs at 900 °C for 108 h.

Figure 4 illustrates the mass gains of FeNiCu and three HEAs oxidized at 900 °C for 108 h. All HEAs exhibit significantly lower total mass gains (<2.6 mg/cm^2^) compared with the FeNiCu alloy (24.4 mg/cm^2^), highlighting the superior oxidation resistance of the FeNiCuAlCr, FeNiCuAlCo, and FeNiCuAlMn HEAs at elevated temperatures. Among the HEAs, FeNiCuAlMn demonstrates a mass gain of 2.55 mg/cm^2^, while FeNiCuAlCo shows a reduction of more than half (1.1 mg/cm^2^). Notably, FeNiCuAlCr exhibits the lowest mass gain (0.83 mg/cm^2^) over 108 h, indicating that the addition of Cr significantly enhances oxidation resistance in HEAs.

Oxidation resistance and thermal stability are crucial factors for high-temperature applications of oxygen-affine alloy systems. Nevertheless, there exists a paucity of research concerning the mechanisms underlying the formation and oxidation behaviors of high-entropy alloys (HEAs) [24]. Oxidation resistance is particularly critical for materials operating under high-temperature conditions. Generally, metal alloy oxidation occurs through two primary mechanisms [25]. The first mechanism involves direct contact between oxygen and the metal surface, where the rate-controlling step is the gas–metal interface reaction. In this scenario, the oxidation growth follows the subsequent specific growth law:

(1) $$\left(\frac{\Delta m}{A}\right)=Kt^{n}+C$$

In this equation, Δ*m*/*A* refers to the mass increase per unit surface area, *K* represents the constant for the oxide growth rate, *t* signifies the duration of the oxidation process, and *n* is the time exponent. *C* refers to the mass change data, the surface area of the sample, the kinetic constant, and the integration constant.

The initial oxidation mechanism involves a linear increase in the mass-to-area ratio over time, with *n* = 1, indicating unity in the oxidation growth equation. This type of oxidation occurs when the oxide layer formed on the surface fails to develop into a continuous, stable passive film. This instability can be attributed to factors such as insufficient oxide volume relative to the alloy volume, the rapid evaporation of oxides with a low vapor pressure, or high oxygen solubility and transport rates within the matrix [26].

The second stage is characterized by the formation of a dense, passive oxide layer on the alloy surface, which serves as a protective barrier against direct exposure to gaseous oxygen. At this stage, diffusion through the oxide layer becomes the rate-limiting factor in the oxidation process. As a result, the rate of mass gain per unit area diminishes with time. In this case, the volume of the oxide layer formed on the surface is equal to or exceeds the volume of the alloy undergoing oxidation, and the oxide exhibits strong adhesion to the substrate. Under such circumstances, the oxidation growth adheres to a parabolic relationship, with a time exponent (*n*) of 0.5.

**Figure 5:**
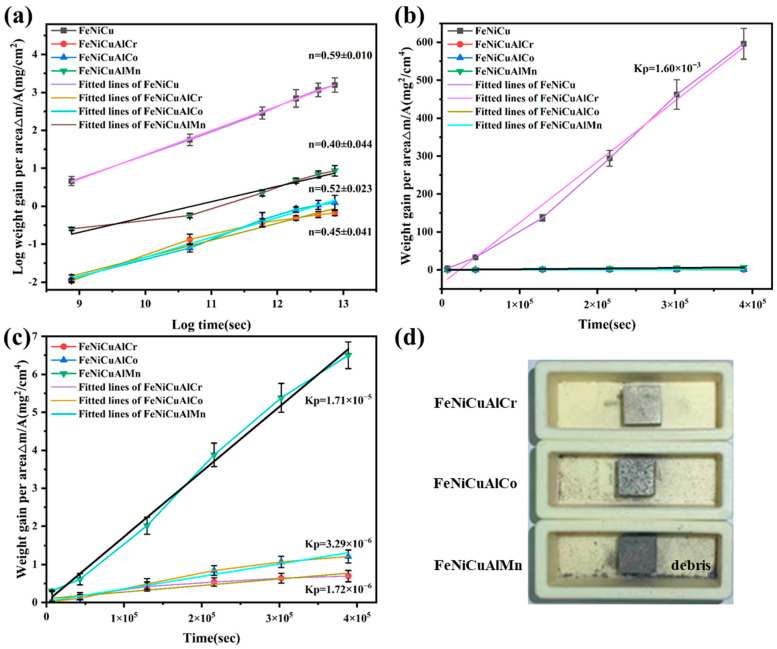
(**a**) Logarithm of mass gain vs. logarithm of oxidation time plots for the FeNiCu alloy and the FeNiCuAlCr, FeNiCuAlCo, and FeNiCuAlMn HEAs at 900 °C; (**b**,**c**) parabolic plots of oxidation kinetics of FeNiCu alloy and FeNiCuAlCr, FeNiCuAlCo, and FeNiCuAlMn HEAs at 900 °C; (**d**) picture after the oxidation experiment.

The double logarithmic plot of mass gain versus exposure time at 900 °C, spanning from 0 to 108 h, is presented in Figure 5a,b. The *n*-values range from 0.40 to 0.59, closely aligning with the theoretical value of 0.50. This finding further confirms that the oxidation kinetics of the alloy and high-entropy alloys (HEAs) follow the parabolic rate law. Additionally, Figure 5c depicts the parabolic plots of oxidation kinetics for the FeNiCu, FeNiCuAlCr, FeNiCuAlCo, and FeNiCuAlMn HEAs at 900 °C. The calculated parabolic rate constants (*kp*), derived from the squares of the slopes via linear least-squares fitting, are summarized in Table 1. Notably, the values of the FeNiCuAlCr, FeNiCuAlCo, and FeNiCuAlMn HEAs are approximately 2–3 orders of magnitude lower than that of the FeNiCu alloy at 900 °C. The *kp* value for the FeNiCuAlCr HEA decreased by 1–2 orders of magnitude to 1.72 × 10^−6^ mg^2^·cm^4^/s compared with the FeNiCuAlCo (3.29 × 10^−6^ mg^2^·cm^4^/s) and FeNiCuAlMn HEAs (1.71 × 10^−5^ mg^2^·cm^4^/s). This demonstrates that the addition of Cr significantly enhances the oxidation resistance of FeNiCuAlCr HEAs. Figure 5d illustrates the physical appearance of the three HEAs after 108 h of oxidation, where it is evident that the high-entropy alloy containing Mn exhibits more pronounced debris detachment.

From a thermodynamic perspective, the Gibbs free energy (ΔG) of metal oxides follows the order Al_2_O_3_ < MnO < Cr_2_O_3_ < Fe oxides < CoO < NiO < 0, indicating that all possible oxides can form spontaneously during the oxidation process. In the FeNiCuAlCr HEA, the affinity of oxygen for Al and Cr is significantly higher, leading to the preferential formation of Al_2_O_3_, which predominantly remains in the outer oxide layer [29]. A transitional phase, typically lasting several hours, is also noted, during which selective nucleation and the subsequent growth of oxides lead to a non-uniform distribution of the oxide layer. During this period, metastable oxides that form either undergo transformation into stable phases or dissolve into other oxide compounds [30]. At the onset of oxidation, the elevated oxygen partial pressure at the alloy surface plays a pivotal role in the interfacial reactions. Oxygen molecules are initially absorbed by the surface, dissociating into oxygen ions (O^2−^), which then react with aluminum to produce a thin layer of aluminum oxide. As oxidation proceeds, surface Al is gradually depleted, and the inherently sluggish diffusion of HEAs diminishes the tendency for selective oxidation [31]. When the Al content in the depletion zone falls below the threshold concentration required for Al_2_O_3_ formation, other oxides such as Cr_2_O_3_, Fe_2_O_3_, and MnO begin to develop. At this stage, diffusion becomes the dominant mechanism. Oxygen migrates inward, while Cr, Fe, Co, Cu, and Mn within the Al-depleted region react with oxygen to form their respective oxides. These oxides may dissolve or react with other intermediate oxides to create a stable spinel phase. In the final phase of oxidation, the oxide film becomes compact and continuous, restricting diffusion. Consequently, the rate of weight gain stabilizes and remains nearly constant.

### 3.3. Oxide Phase Constituents

**Figure 6:**
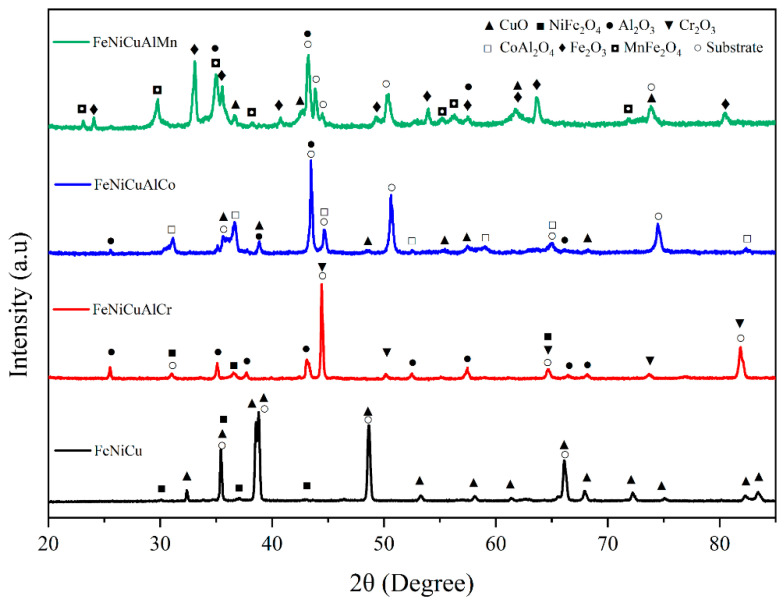
XRD patterns of HEAs after oxidation at 900 °C for 108 h.

The XRD patterns of the FeNiCu, FeNiCuAlCr, FeNiCuAlCo, and FeNiCuAlMn high-entropy alloys (HEAs) after oxidation are shown in Figure 6. The spectrum of the FeNiCu alloy reveals that the oxide scale primarily consists of CuO along with spinel-type oxides (NiFe_2_O_4_, as confirmed in the Supplementary Figure S2 and Table S1). All three HEAs were found to contain Al_2_O_3_. In addition to Al_2_O_3_, the oxide scale of the FeNiCuAlCr HEA is predominantly composed of Cr_2_O_3_ and spinel-type oxides, whereas the oxide scale of the FeNiCuAlCo HEA is mainly composed of CuO and spinel-type oxides. The oxide scale of the FeNiCuAlMn HEA is primarily made up of CuO, Fe_2_O_3_, and spinel-type oxides, with the spinel-type oxides identified as NiFe_2_O_4_, CoAl_2_O_4_, and MnFe_2_O_4_, respectively. The formation of protective Cr_2_O_3_ and Al_2_O_3_ may explain the superior oxidation resistance of the FeNiCuAlCr HEA.

### 3.4. Microstructure and Chemical Composition of the Oxide Scale

**Figure 7:**
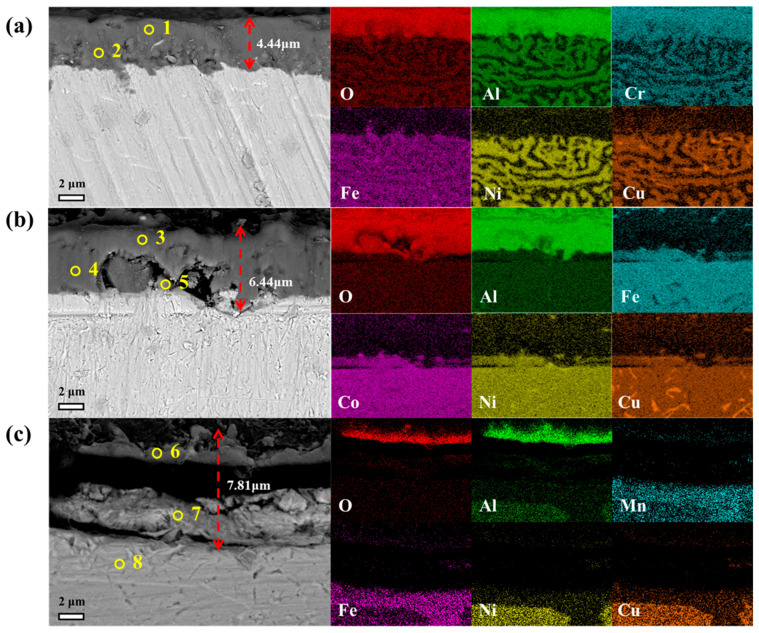
BSE-SEM and EDS images of (**a**) FeNiCuAlCr, (**b**) FeNiCuAlCo, and (**c**) FeNiCuAlMn HEAs after oxidation for 108h at 900 °C.

Figure 7 presents the cross-sectional energy-dispersive X-ray spectroscopy (EDS) mappings along with the corresponding backscattered electron (BSE) micrographs of the oxide scale formed on each tested sample. Panels (a), (b), and (c) correspond to the FeNiCuAlCr, FeNiCuAlCo, and FeNiCuAlMn high-entropy alloys (HEAs), respectively. Based on the distribution of oxygen and the BSE images, it is evident that the FeNiCuAlCr HEA exhibits the thinnest oxide scale (~4.44 µm), which is consistent with the lowest mass gain observed in Figure 4.

The formation of multiple oxide scales is confirmed through EDS mappings and point analysis (Table 2). The Al-, Cr-, and Fe-rich scales on the FeNiCuAlCr HEA correspond to the oxides Al_2_O_3_, Cr_2_O_3_, and NiFe_2_O_4_, as observed in Figure 7a. This indicates that the inner oxide layer consists of NiFe_2_O_4_, while the outer oxide layer is composed of Al_2_O_3_ and a mixture of C_2_O_3_. The oxide scale of the FeNiCuAlCo HEA is thicker than that of the FeNiCuAlCr HEA, measuring 6.13 μm. The EDS mappings in Figure 7b confirm the presence of externally oxidized Al and the formation of an Al_2_O_3_ oxide scale. The atomic ratio of the Cu-rich particles (point 4) is consistent with CuO, while the (Co, Fe)-rich particles (point 5) are similar to NiFe_2_O_4_ and CoAl_2_O_4_. Figure 7c shows the triplex oxide scale formed on the FeNiCuAlMn HEA. Based on the combined XRD and EDX mapping results, it can be concluded that the outer oxide layer is primarily composed of Al_2_O_3_. The central oxide layer, identified by the (Fe, Mn)-rich region (point 7), contains Fe_2_O_3_ and MnFe_2_O_4_. Additionally, the (Fe, Ni, Cu, Al, Mn)-rich region (point 8) exhibits a contrast similar to the substrate.

**Figure 8:**
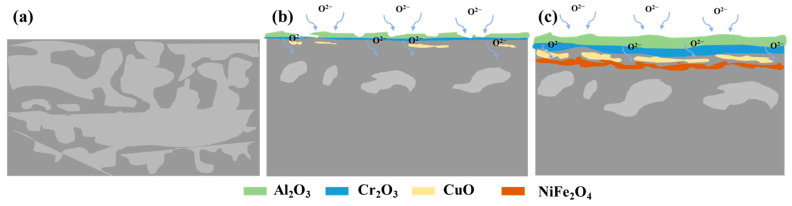
Schematic diagram of the oxidation mechanism of FeNiCuAlCr alloy at 900 °C in the air: (**a**) Unoxidized sample; (**b**) Early stage of oxidation and (**c**) Final stage of oxidation.

As observed during the isothermal oxidation process, the outer oxide layers of the FeNiCuAlCo and FeNiCuAlMn HEAs develop wrinkles, while the inner oxide layers bend and may even detach from the substrate (Figure 7b,c), leading to the formation of an irregular oxide layer. In contrast, the oxide film of the FeNiCuAlCr HEA is denser and remains free of cracks. Figure 8 is a schematic of the oxidation mechanism of FeNiCuAlCr alloys in air at 900 °C. In the oxidation process of FeNiCuAlCr high-entropy alloys, Al_2_O_3_ initially forms on the surface, where it acts as a passivating layer, preventing further oxygen diffusion and thereby limiting the oxidation of internal elements. Subsequently, chromium oxide and copper oxide layers form. As the oxidation progresses, the Al_2_O_3_ and Cr_2_O_3_ oxide layers grow thicker and merge, with oxygen gradually migrating inward. Over time, under high-temperature oxidation conditions, the system reaches local equilibrium in oxygen partial pressure, leading to the formation of continuous spinel-type oxides (NiFe_2_O_4_) due to reactions between Ni and Fe oxides. These factors contribute to the excellent high-temperature oxidation resistance of FeNiCuAlCr alloys.

The superior oxidation resistance of the FeNiCuAlCr HEA, along with the mechanisms underlying the formation of wrinkles and cracks in the FeNiCuAlCo and FeNiCuAlMn HEAs, will be discussed in Section 3.6.

### 3.5. Soft X-Ray XANES

Although the SEM and EDS images confirmed the formation of oxides on the surfaces of the high-entropy alloys (HEAs) during isothermal oxidation, XANES analysis using soft X-rays at the L_2,3_ absorption edges of 3d elements was also employed to investigate changes in the valence states of surface elements in HEAs.

**Figure 9:**
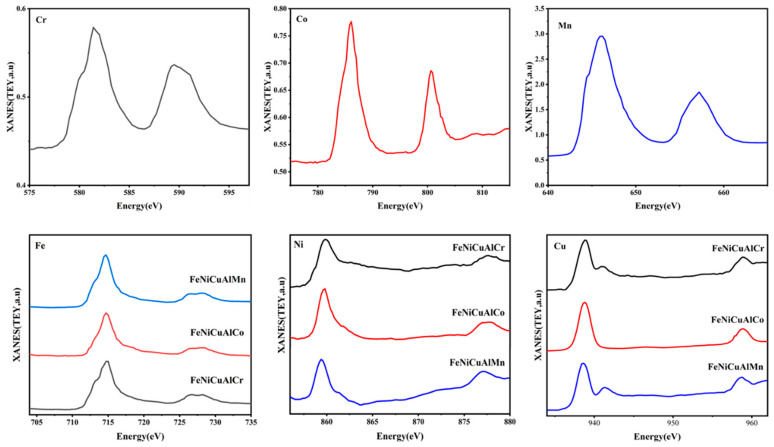
XANES spectra recorded at the L_2,3_ absorption edges of Cr, Co, Mn, Fe, Ni, and Cu.

The raw data, presented in Figure 9, reveal varying degrees of surface oxidation for each element in the individual samples. Interestingly, despite the homogeneous local environment demonstrated by sXAS, the HEAs exhibited the partial oxidation of Co and Cu, with Ni atoms remaining predominantly in a metallic-like state. In contrast, the Cr, Fe, and Mn atoms were significantly more oxidized. Furthermore, the XANES spectra at the Cr L_2,3_ absorption edge display a characteristic shape corresponding to Cr^3+^ ions in Cr_2_O_3_ oxide.

### 3.6. Crack Formation Mechanism of Oxide Scale

The oxidation resistance of an alloy is predominantly governed by the rate of oxidation and the structural integrity of the oxide layer. Once the oxide scale detaches, oxidation accelerates, forming a new oxide layer on the alloy surface. If this new oxide exhibits poor oxidation resistance, the protective performance of the alloy deteriorates [32]. Thus, the integrity of the oxide scale plays a critical role in determining the alloy’s protective performance, which is closely related to the mechanical properties of the oxide layer. Internal stress is a key factor influencing the protective performance of the oxide scale [33]. Stress generated within the oxide layer often leads to cracking and spallation.

To better analyze the peeling phenomenon, the Pilling–Bedworth Ratio (PBR) is utilized to assess the contribution of individual oxides to the development of internal stress. The PBR is a crucial metric for assessing the structural integrity of the oxide scale. It represents the ratio of the oxide volume to the volume of metal consumed during oxide formation and is heavily influenced by the alloy composition [34]. When the PBR exceeds 1, compressive stress develops within the oxide film. Notably, when the PBR surpasses 3, excessive compressive stress can cause the oxide film to wrinkle and delaminate. The PBR can be calculated using the following formula [35]:

(2) $$PBR=\frac{V_{ox}}{V}$$

(3) $$V_{ox}=\frac{M_{ox}}{{x\rho }_{ox}}$$

(4) $$V=\frac{A_{a}}{\rho_{a}}$$

In this context, *ρ_ox_* refers to the density of the oxide, while *ρ_a_* indicates the density of the alloy at ambient temperature. *A_a_* is defined as (1 − a − … − b) MA + … + b MB + a MC (at%), representing the effective atomic mass of the alloy. x corresponds to the number of metal atoms in the respective oxide. The physical constants and calculated results are provided in Table 3. The PBR values for all oxides are found to range between 1 and 3; this suggests that the oxide scale has reached a sufficient thickness to completely cover the alloy surface, while simultaneously maintaining an acceptable range of internal stresses, which are primarily compressive in nature [36]. For FeNiCuAlMn, the PBR values of MnFe_2_O_4_ and Fe_2_O_3_ are 2.2 and 2.28, respectively, suggesting a higher propensity for the oxide scale to delaminate. This observation aligns with the oxidation weight gain curve in Figure 4 and is further supported by the results from the actual picture (a huge crack occurred between point six and point seven in Figure 5d). In the physical images after the oxidation experiment, we observed that although the surface oxide layer of FeNiCuAlCr high-entropy alloy experienced less spallation compared with the FeNiCuAlCo and FeNiCuAlMn high-entropy alloys, there was still some minor spallation. This could be attributed to the difference in the thermal expansion coefficients between the metal oxide and the alloy. After the isothermal oxidation experiment, all three high-entropy alloys formed dense Al_2_O_3_ or a combination of Al_2_O_3_ and Cr_2_O_3_ oxide films on their surfaces. Since the thermal expansion coefficient of the oxide film is typically lower than that of the alloy itself, this difference could generate stresses at the oxide–metal interface, leading to the spallation of the oxide layer [37,38].

### 3.7. Corrosion Behavior

**Figure 10:**
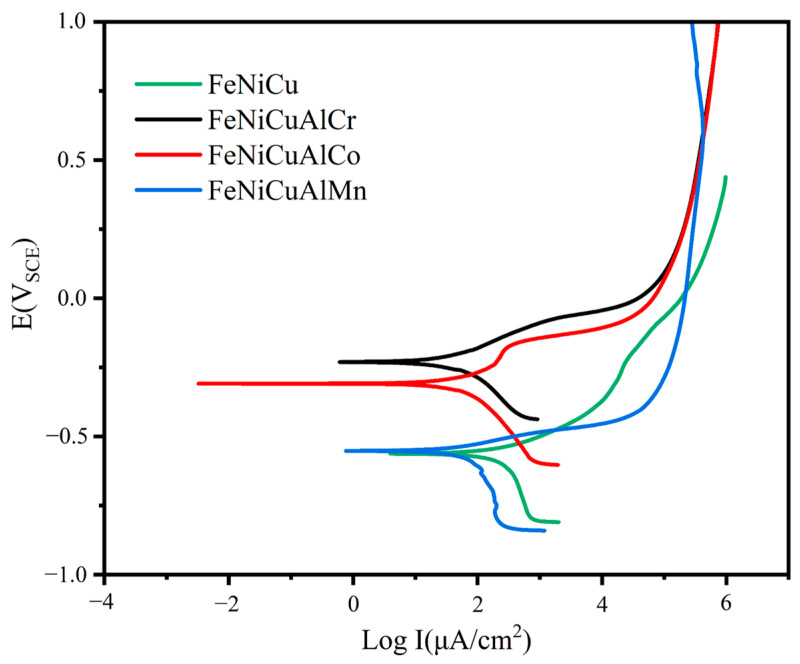
Potentiodynamic polarization curves of the FeNiCu, FeNiCuAlCr, FeNiCuAlCo, and FeNiCuAlMn HEAs in the 3.5 wt% NaCl solution.

To investigate the corrosion behavior of the alloys, a potentiodynamic polarization test was conducted in a deaerated 3.5 wt% NaCl aqueous solution (as shown in Figure S3, where the open circuit potential of all samples was stabilized prior to testing), with the resulting potentiodynamic curves shown in Figure 10. A distinct passivation region is visible in the curves. The Tafel extrapolation method was employed to derive corrosion parameters [39]. The self-corrosion potential (*E_corr_*) and corrosion current density (*i_corr_*) were determined from the point of intersection between the linear anodic and cathodic branches of the polarization curves. To ensure accurate extrapolation, at least one of the branches must adhere to the Tafel relationship, which is linear on a semilogarithmic scale, over a current density range spanning one decade, with extrapolation starting 50–100 mV away from *E_corr_*. The corrosion parameters derived are summarized in Table 4. Higher *E_corr_* and lower *i_corr_* values indicate greater stability and corrosion resistance in saline solutions [40,41,42]. The corrosion current densities for the FeNiCu, FeNiCuAlCr, FeNiCuAlCo, and FeNiCuAlMn alloys in 3.5 wt% NaCl solution were found to be 240 μA/cm^2^, 45.7 μA/cm^2^, 81.3 μA/cm^2^, and 85.1 μA/cm^2^ respectively. Furthermore, the *E_corr_* value of the FeNiCuAlCr HEA was the highest among the alloys at −0.21 V, demonstrating that the FeNiCuAlCr HEA exhibits the best corrosion resistance in a 3.5 wt% NaCl solution.

**Figure 11:**
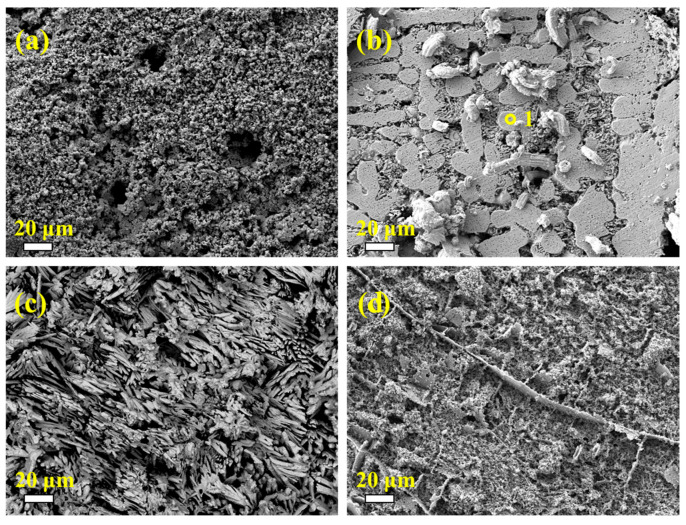
The corrosion surface morphologies of samples after potentiodynamic polarization in 3.5% NaCl aqueous solution: (**a**) FeNiCu; (**b**) FeNiCuAlCr; (**c**) FeNiCuAlCo; (**d**) FeNiCuAlMn.

It is noteworthy that the Cr-containing high-entropy alloys exhibit strong corrosion resistance, which may be attributed to the formation of a FeCr-rich BCC phase within the alloy. Zemanate et al. studied the corrosion performance of AlCoCrFeNix high-entropy alloys with different Ni concentrations using potentiodynamic polarization in a 3.5% NaCl solution. They found that the Cr-rich BCC phase showed better corrosion resistance. Similarly, Cui et al. discovered that in the Fe_1.125_Ni_1.06_CrAl high-entropy alloy, the BCC phase rich in Fe and Cr elements was resistant to corrosion and remained intact. The scanning images after potentiodynamic polarization (Figure 11) reveal that corrosion starts at the grain boundaries. Except for the FeNiCuAlCr high-entropy alloy, the other three samples exhibited severe corrosion, with FeNiCuAlCr showing better corrosion resistance compared with the others. The EDS data in Table 4 indicates that the areas not severely corroded, such as point one, are primarily composed of the FeCr-rich phase, which is consistent with previous studies.

**Figure 12:**
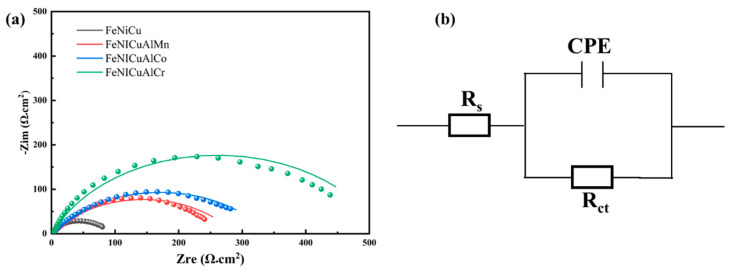
(**a**) Nyquist plots. (**b**) Equivalent electrical circuit for fitting the EIS data of the HEAs at the open circuit potential in the 3.5 mass% NaCl solution.

Electrochemical impedance spectroscopy (EIS) was also employed to study the formation and behavior of the passive layer in the corrosive medium [43]. Figure 12 presents the Nyquist plots of the alloys in the 3.5 wt% NaCl solution at open circuit potential. The semicircle radii of the Nyquist plots for FeNiCu, FeNiCuAlMn, FeNiCuAlCo, and FeNiCuAlCr alloys increase in that order, indicating a progressive enhancement of impedance in the passive films. This trend suggests that the addition of Cr improves the corrosion resistance of the alloys, consistent with the immersion test results and potentiodynamic polarization analysis.

The *Rs* (*Rct* CPE) model [44] was used to fit the EIS spectra of the alloys. In this model, *R_s_* represents the solution resistance, *R_ct_* corresponds to the charge transfer resistance (reflecting the corrosion rate of the metal in the solution), and the constant phase element (CPE) accounts for surface inhomogeneity and adsorption effects. The impedance of CPE (*Z_CPE_*) is defined by

(5) $$Z_{CPE}=Y_{0}^{-1}{\left(j\omega \right)}^{-\alpha }$$

where *Y*_0_ is the proportionality constant, *j* is the imaginary unit, ω is the angular frequency, and α (ranging from −1 to 1) is the phase exponent that reflects deviations from ideal capacitive behavior. For α = 1, the CPE acts as a pure capacitor; for α = 0, it behaves as a resistor; and for α = −1 it exhibits inductive characteristics [45].

The equivalent circuit depicted in Figure 12b was applied to fit the EIS data, with the fitting parameters summarized in Table 5. Through EIS fitting, we obtained the Rct value for the Cr-containing alloys, which reflects the ease with which charge transfers from the alloy to the electrolyte. A higher Rct value indicates that the transfer of electrons from the metal to the electrolyte is more difficult, suggesting that the alloy exhibits better corrosion resistance. Corrosion resistance is typically associated with the passive layer on the metal surface, which prevents further interaction between the metal and the corrosive medium. Cr-containing alloys can form a stable oxide film, which plays a protective role and suppresses the corrosion reaction. In EIS, a high Rct value indicates that the charge transfer process is hindered, demonstrating the significant role the passive layer plays in protecting the alloy. For Cr-containing alloys, their higher Rct values and good fitting of the CPE further support their strong corrosion resistance. The EIS results are consistent with those obtained from the potentiodynamic polarization curves. In the polarization tests, the corrosion potential, corrosion current density, and the size of the passive region reflect the corrosion resistance of the alloy. For Cr-containing high-entropy alloys, the potentiodynamic polarization curve shows a larger passive region, which means the alloy surface can maintain a stable passive state in the corrosive medium, reducing the likelihood of corrosion.

Additionally, the superior corrosion resistance of Cr-containing high-entropy alloys may be attributed to their lower double-layer capacitance values. According to the empirical equation proposed by Mansfeld et al., we will use the following formula to calculate the double-layer capacitance (Cdl):

(6) $$Cdl=Q_{0}{\left(\omega_{max}\right)}^{n-1}$$

where *Q*_0_ is the CPE parameter, *ω_max_* is the frequency at which the imaginary part of impedance is maximum, and n is the exponent of the CPE.

A smaller double-layer capacitance value indicates that the Cr-containing high-entropy alloys form a more uniform and defect-free passive film, which effectively protects the substrate. A series of results demonstrate that the FeNiCuAlCr high-entropy alloy exhibits excellent corrosion resistance in 3.5% NaCl solution.

## 4. Conclusions

In this study, the oxidation behavior of the FeNiCuAlCr, FeNiCuAlCo, and FeNiCuAlMn high-entropy alloys (HEAs) was investigated in air at 900 °C, and their corrosion resistance in 3.5% NaCl solution was evaluated using potentiodynamic polarization and electrochemical impedance spectroscopy (EIS). The following conclusions were drawn:

(1) The oxidation kinetics of the FeNiCuAlCr, FeNiCuAlCo, and FeNiCuAlMn HEAs in air at 900 °C follow a parabolic relationship. Among them, the FeNiCuAlCr HEA exhibited excellent oxidation resistance, with a low mass gain of 0.83 mg/cm^2^ after 108 h of exposure at 900 °C.
(2) The outer oxidation layer of the FeNiCuAlCr HEA formed a dense and continuous Al_2_O_3_ and Cr_2_O_3_ layer, which provided effective protection for the substrate. In contrast, for the FeNiCuAlCo and FeNiCuAlMn HEAs, prolonged oxidation and increased high-temperature internal stress led to the partial peeling and wrinkling of the oxide scale at the interface between the scale and the alloy.
(3) The electrochemical analysis revealed that the FeNiCuAlCr HEA possesses a high corrosion potential (*E_corr_* = −0.21 V) and a low corrosion current density (*I_corr_* = 45.7 μA/cm^2^). The EIS results further confirmed the superior performance of the passive film, with a high polarization resistance (*R_ct_* = 519.2 Ω cm^2^), indicating excellent stability and protective efficacy.

This study provides new perspectives and a new understanding of the oxidation and corrosion behaviors of high-entropy alloys, revealing their outstanding performance during the oxidation process. With the development of materials science, high-entropy alloys, as a novel material, possess great potential in industrial applications due to their superior mechanical properties, high-temperature resistance, and corrosion resistance. In the future, with continuous research, it is expected that high-entropy alloys will be widely applied in various fields such as aerospace, energy, chemical engineering, and automotive manufacturing. Especially in high-temperature, high-pressure, and extreme environments, they are expected to become important structural and functional materials.

## Figures and Tables

**Table 1:** Fitting parameters (*Kp* and *n*) for the oxidation of FeNiCuAlCr, FeNiCuAlCo, FeNiCuAlMn HEAs oxidized at 900 °C for 108 h.

HEAs	*Kp* (mg^2^·cm^4^/s)	*n*
FeNiCu	1.60 × 10^−3^	0.59
FeNiCuAlCr	1.72 × 10^−6^	0.40
FeNiCuAlCo	3.29 × 10^−6^	0.52
FeNiCuAlMn	1.71 × 10^−5^	0.45
Al_15_CoCrFeNi [27]	4.7 × 10^−5^	
FeMnCrCoNi [28]	4.1 × 10^−5^	

**Table 2:** Chemical compositions of the yellow point regions in Figure 5 (at%).

Region	Fe	Ni	Cu	Al	Cr	Co	Mn	O
1	0.27	-	-	24.95	13.49	-	-	61.29
2	6.80	2.94	1.22	20.81	9.32	-	-	58.91
3	0.28	0.08	0.13	41.24	-	0.11	-	58.16
4	0.13	0.72	4.42	36.33	-	1.41		56.99
5	1.12	0.62	2.70	33.42	-	7.32		54.82
6	0.11	0.00	0.02	33.96	-	-	0.37	65.54
7	16.35	9.11	10.32	7.11	-	-	13.07	44.04
8	19.45	13.00	13.09	10.11	-	-	18.22	26.13

**Table 3:** The calculated PBR values of the main oxides in the FeNiCuAlCr, FeNiCuAlCo, and FeNiCuAlMn HEAs.

	CuO	Al_2_O_3_	Cr_2_O_3_	Fe_2_O_3_	NiFe_2_O_4_	CoAl_2_O_4_	MnFe_2_O_4_
FeNiCuAlCr	1.89	2.17	2.16	-	2.16	-	-
FeNiCuAlCo	1.96	2.16	-	-	-	2.09	-
FeNiCuAlMn	1.89	2.16	-	2.20	-	-	2.28

**Table 4:** Chemical compositions of the yellow point regions in Figure 10 (at%).

Region	Fe	Ni	Cu	Al	Cr	Na	Cl	O
1	31.10	11.11	4.25	8.23	30.51	0.09	3.70	11.01

**Table 5:** Electrochemical parameters of the alloys in the 3.5 mass% NaCl solution.

Alloy	E_corr_ (V)	I_corr_ (μA/cm^2^)	Rs (Ω cm^2^)	α	Rct (Ω cm^2^)
FeNiCu	−0.57	240	0.82	0.72	89.61
FeNiCuAlCr	−0.21	45.7	1.53	0.76	519.2
FeNiCuAlCo	−0.34	81.3	1.56	0.64	338.7
FeNiCuAlMn	−0.56	85.1	1.39	0.63	286.2

## Data Availability

The original contributions presented in this study are included in the article/supplementary material. Further inquiries can be directed to the corresponding authors.

## Supplementary Materials

The following supporting information can be downloaded at: https://www.mdpi.com/article/10.3390/ma18153679/s1. Figure S1: SEM image(BSE) of FeNiCuAl as-cast HEAs; Figure S2: BSE-SEM and EDS images of FeNiCu alloys; Figure S3: The open circuit potential diagrams of the four high-entropy alloys in 3.5% sodium chloride solution; Table S1: Chemical compositions of the yellow point regions in Figure S1 (at%).
