# Supplementary material for: Unraveling the Excellent High-Temperature Oxidation Behavior of FeNiCuAl-Based Alloy
Source: Materials (Basel). 2025 Aug 5;18(15):3679. doi: 10.3390/ma18153679 (PMC12348563; doi:10.3390/ma18153679)
Supplement: Supplementary file 1 [file materials-18-03679-s001.zip › materials-3751948-supplementary.pdf]

Supplementary Materials

Unraveling the Excellent High-Temperature Oxidation Behavior of FeNiCuAl-Based Alloy

Guangxin Wu [1], Gaosheng Li [2], Lijun Wei [2], Hao Chen [1], Yujie Wang [1], Yunze Qiao [1], Yu Hua [1], Chenyang Shi [1], Yingde Huang [1,*] and Wenjie Yang [1,*]

[1] School of Materials Science and Engineering, Zhengzhou University, Zhengzhou 450001, China
[2] State Power Investment Corporation Limited Central Research Institute, Beijing 102209, China
* Correspondence: ydhuang@zzu.edu.cn (Y.H.); ywj2012@zzu.edu.cn (W.Y.)

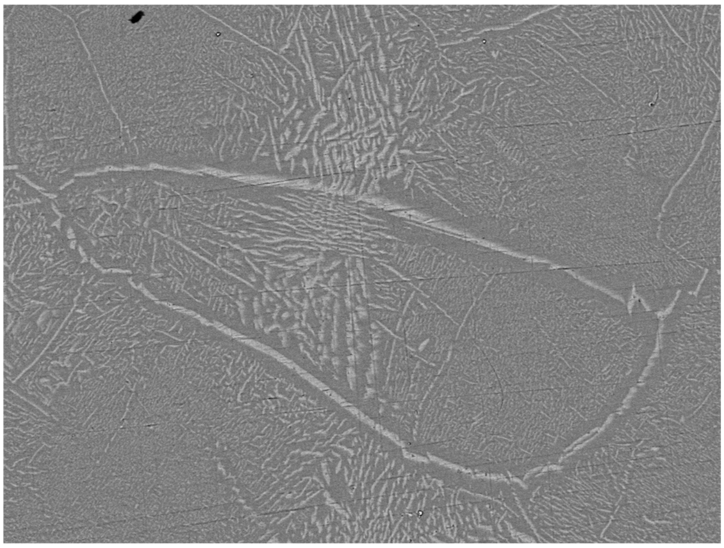

Figure S1. SEM image(BSE) of FeNiCuAl as-cast HEAs.

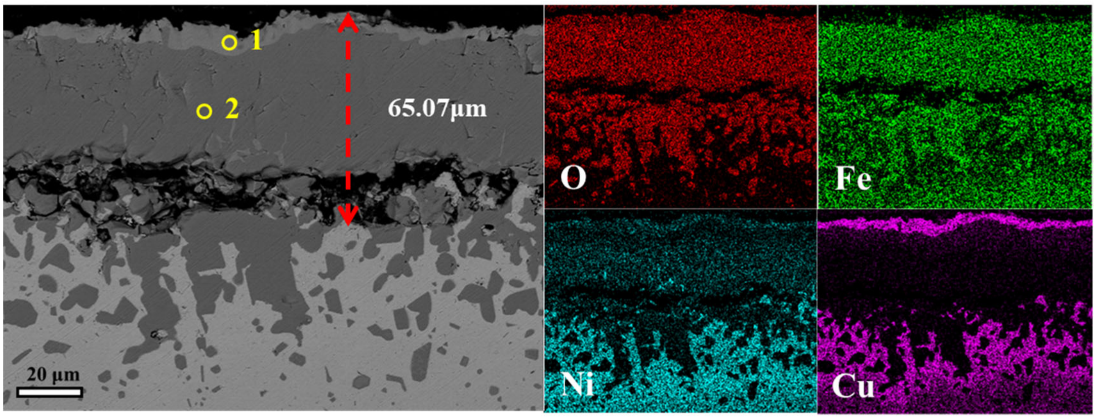

Figure S2. BSE-SEM and EDS images of FeNiCu alloys.

Table S1. Chemical compositions of the yellow point regions in Figure S1 (at%).

Region	Fe	Ni	Cu	O
1	0.10	0.16	49.20	50.54
2	28.19	15.27	0.23	56.31

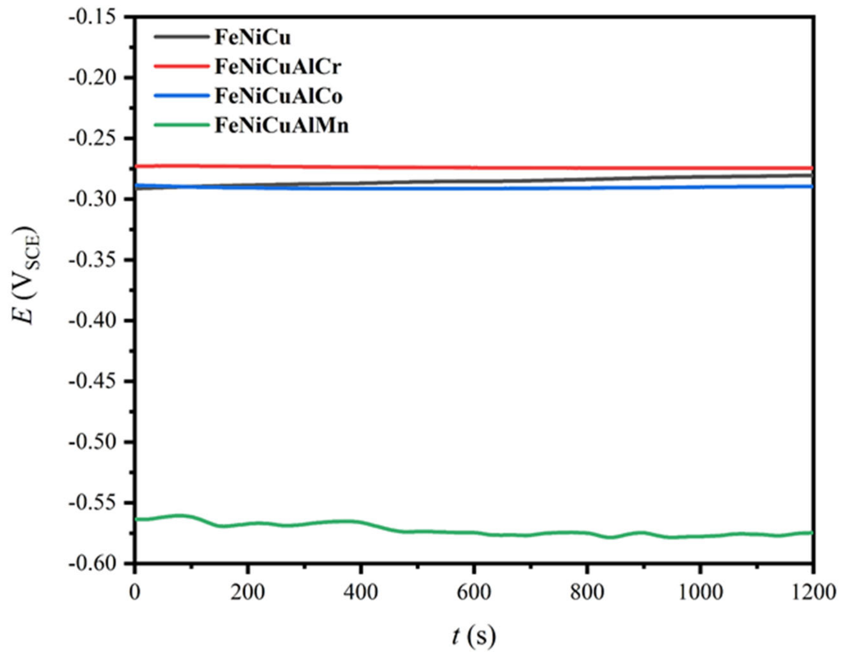

Figure S3. The open circuit potential diagrams of the four high-entropy alloys in 3.5% sodium chloride solution.
